# Sky1 regulates the expression of sulfur metabolism genes in response to cisplatin

**DOI:** 10.1099/mic.0.078402-0

**Published:** 2014-07

**Authors:** Silvia Rodríguez-Lombardero, Ángel Vizoso-Vázquez, Luis J. Lombardía, Manuel Becerra, M. Isabel González-Siso, M. Esperanza Cerdán

**Affiliations:** 1 Grupo EXPRELA, Departamento de Bioloxía e Celulare Molecular, Facultade de Ciencias, Universidade da Coruña, Campus de A Coruña, 15071 A Coruña, Spain; 2 Centro Nacional de Investigaciones Oncológicas (CNIO), C/Melchor Fernández Almagro 3, 28029 Madrid, Spain

## Abstract

Cisplatin is commonly used in cancer therapy and yeast cells are also sensitive to this compound. We present a transcriptome analysis discriminating between RNA changes induced by cisplatin treatment, which are dependent on or independent of *SKY1* function – a gene whose deletion increases resistance to the drug. Gene expression changes produced by addition of cisplatin to W303 and W303-Δ*sky1* cells were recorded using DNA microarrays. The data, validated by quantitative PCR, revealed 122 differentially expressed genes: 69 upregulated and 53 downregulated. Among the upregulated genes, those related to sulfur metabolism were over-represented and partially dependent on Sky1. Deletions of *MET4* or other genes encoding co-regulators of the expression of sulfur-metabolism-related genes, with the exception of *MET28*, did not modify the cisplatin sensitivity of yeast cells. One of the genes with the highest cisplatin-induced upregulation was *SEO1*, encoding a putative permease of sulfur compounds. We also measured the platinum, sulfur and glutathione content in W303, W303-Δ*sky1* and W303-Δ*seo1* cells after cisplatin treatment, and integration of the data suggested that these transcriptional changes might represent a cellular response that allowed chelation of cisplatin with sulfur-containing amino acids and also helped DNA repair by stimulating purine biosynthesis. The transcription pattern of stimulation of sulfur-containing amino acids and purine synthesis decreased, or even disappeared, in the W303-Δ*sky1* strain.

## Introduction

Cisplatin (*cis*-diaminodichloroplatinum) is commonly used in cancer therapy. The molecular mechanisms of cisplatin cytotoxicity include the binding of the drug to DNA and non-DNA targets, and the subsequent induction of cell death. The binding of cisplatin to DNA may interfere with normal transcription and/or DNA replication mechanisms that would trigger the cytotoxic effect. However, it is known that only 5–10 % of covalently bound cisplatin is found in the DNA fraction, whereas the rest interacts with proteins and other molecules (Basu & Krishnamurthy, 2010; Fuertes *et al.*, 2003). Cisplatin resistance is also a multi-factorial process, which might be influenced by changes in mismatch repair mechanisms or cell signalling, increased cisplatin detoxification, or failure to undergo apoptosis, among others (Basu & Krishnamurthy, 2010; Pérez, 1998).

*Saccharomyces cerevisiae* has been widely used as a powerful eukaryotic model to find genes related to cisplatin sensitivity (Burger *et al.*, 2000; Wu *et al.*, 2004) or cisplatin resistance (Huang *et al.*, 2005; Wu *et al.*, 2004) because libraries of mutant strains containing single deletions of all the genes present in this yeast genome are available. Frequently, genes identified in yeast and human models are coincident. This is the case with *SKY1* and *SRPK1*, which encode protein-specific serine–arginine (SR) kinases related to the cytotoxicity caused by cisplatin in yeast and human, respectively (Schenk *et al.*, 2001). *SRPK1* shows 53 % sequence similarity and is a functional homologue of *SKY1*, as shown by heterologous complementation of the cisplatin-resistant phenotype of a Δ*sky1* strain (Schenk *et al.*, 2001).

Interestingly, the responses to cisplatin and to a *SKY1* deletion both involve alterations in DNA repair pathways (Schenk *et al.*, 2002). Among the known targets of Sky1 are proteins related to mRNA export from the nucleus (Gilbert *et al.*, 2001; Windgassen & Krebber, 2003; Windgassen *et al.*, 2004) and RNA splicing (Dagher & Fu, 2001; Shen & Green, 2006). Therefore, important changes in cellular mRNA levels as a consequence of *SKY1* deletion should be expected.

Recently, the potential of transcriptome analyses to compare gene expression profiles under different conditions has been applied to find specific patterns related to cisplatin sensitivity (Cavill *et al.*, 2011; Wan *et al.*, 2010) or cisplatin resistance (Kim *et al.*, 2011; Wan *et al.*, 2010) in cancerous cells. The major advantage of using the yeast model for transcriptome analyses is that it allows the easy and simultaneous testing of cisplatin treatment and the loss of function of a specific gene. In this work, we present a genome-wide transcriptional study of *S. cerevisiae* W303 and W303-Δ*sky1* cells in the presence or absence of cisplatin. The effects of cisplatin treatment were further validated by quantitative (q)PCR. The functional significance of the transcriptome response is discussed with reference to the platinum, sulfur and glutathione content, and related to the results of cytotoxicity assays.

## Methods

### 

#### Strains.

The *S. cerevisiae* strain W303 (*MATa ade2-1 can1-100 his3-11,15 leu2-3,112 trp1-100 ura3-52*) and its derivative W303-Δ*sky1* have been described previously (Rodríguez Lombardero *et al.*, 2012) and were used in these analyses. The *S. cerevisiae* strains yMT-1465 (*ade2-1 can1-100 his3-1,15 leu2-3,112 trp1-1 ura3 met31* : : *TRP1 met32* : : *HIS3*), yMT1782 (*ade2-1 can1-100 his3-1,15 leu2-3,112 trp1-1 ura3 met28* : : *LEU2*), yMT1813 (*ade2-1 can1-100 his3-1,15 leu2-3,112 trp1-1 ura3-52met4* : : *TRP1*) and yMT1947 (*ade2-1 can1-100 his3-1,15 leu2-3,112 trp1-1 ura3 cbf1* : : *TRP1*) were also W303 derivatives and have been described previously (Lee *et al.*, 2010).

The knockout strain W303-Δ*seo1* was obtained by one-step replacement with the *LEU2* marker. The plasmid YCplac111 (Gietz & Akio, 1988) was used as a template to amplify a linear fragment containing the *LEU2* gene and two flanking regions of homology to the ORF ends of *SEO1* by PCR using the primers AVV199 (ATGTATTCAATTGTTAAAGAGATTATTGTAGATCCGCCACGACTCATCTCC) and AVV200 (TTATTTTTCATCAGATACTGATAAGGTTTCAACGTCGGTTCCGCGCACATTTCC). After transformation of the W303 strain with the amplified fragment, cells were selected in complete media without leucine. The correct replacement in the *S. cerevisiae* genome was verified by PCR as described previously (Tizón *et al.*, 1999) using primers designed inside the *LEU2* ORF and the flanking regions of *SEO1* external to the recombination event.

#### Cell culture and treatments.

The handling of yeast cells was carried out according to standard procedures. Three biological replicates of cultures and treatments were run. The yeast cells were pre-cultured overnight in 10 ml complete synthetic medium (SD) prepared as described by Zitomer & Hall (1976). The following day, the cells were inoculated at an initial OD_600_ 0.4 in 70 ml SD and grown in 250 ml Erlenmeyer flasks at 30 °C and 250 r.p.m. When cells reached OD_600_ 0.6, the cultures from each strain were divided into two aliquots of 25 ml (control and cisplatin treatment). Cisplatin was added to the treated cultures at a final concentration of 600 µM. The treatment was done at 30 °C and 250 r.p.m. during 4 h in darkness. The concentration of cisplatin and the time-course of the treatment were established previously in trial experiments. Under the selected conditions, cell survival was >80 % and an increase of 15 % survival was observed in the W303-Δ*sky1* strain versus the W303 strain.

#### RNA preparation and microarray analysis.

Three biological replicates of cultures and cisplatin treatments were run. RNA was extracted from a number of cells corresponding to OD_600_ 3 with the Aurum Total RNA Mini kit (Bio-Rad) following the manufacturer’s instructions. Concentration and purity of RNA were evaluated by measuring the ratio *R* = *A*_260_/*A*_280_ (always in the range 1.7<*R*<2.1). RNA integrity was evaluated by the RIN parameter (RNA integrity number) with the 2100 Bioanalyser (Agilent Technologies) according to the manufacturer’s instructions. RIN was ~9 in all the samples.

Twelve GeneChip Yeast Genome 2.0 arrays from Affymetrix were used and processed in the GeneChip system with Autoloader from Affymetrix. We started from 100 ng total RNA from each sample for successive cDNA, antisense RNA generation, labelling with biotin and fragmentation using the GeneChip 3′ IVT Express kit. RNA fragmentation was monitored with the 2100 Bioanalyser, selecting conditions producing fragments from 35 to 200 nt with the majority 100–120 nt. Hybridization, washes and staining were done with the GeneChip HT Hybridization, Wash and Stain kit (Ambion). These kits included RNA poly-A controls (*lys*, *phe*, *thr* and *dap*) from *Bacillus subtilis* to monitor the target labelling process, and they served as sensitivity indicators of target preparation and labelling efficiency. They also included the hybridization controls, which comprise a mixture of biotinylated and fragmented RNA of *bioB*, *bioC* and *bioD* (genes from the biosynthesis of biotin in *Escherichia coli*) and *Cre* (recombinase from bacteriophage P1). These controls monitored the hybridization, washing and staining steps. Control oligo B2 was included to provide alignment signals for image analysis. Image capture and preliminary data analysis were carried out with Affymetrix Expression Console software (v.1.1).

#### Statistical data analysis and data mining.

Array data were normalized and summarized using the rma algorithm from Affymetrix. The data were analysed using the web suite Babelomics (version 4.3) (Medina *et al.*, 2010). Statistical analyses to identify differentially expressed genes (DEGs) were performed by using the LIMMA test (Smyth, 2005), a moderated *t*-test. The false discovery rate was estimated to correct values for multiple comparison (Benjamini & Hochberg, 1995) using an adjusted *P* = 0.05.

Gene descriptions and comparative analyses of lists from DEGs were obtained through Yeast Mine (http://yeastmine.yeastgenome.org/yeastmine/begin.do). The functional distribution of genes in the differentially regulated clusters was analysed using FunSpec (http://funspec.ccbr.utoronto.ca/) developed by Robinson *et al.* (2002). The MIPS Functional Catalogue Database (FunCatDB) was used in the analyses (http://mips.helmholtz-muenchen.de/proj/funcatDB/). For these analyses, a *P*<0.01 was selected. In the report of the analyses carried out with FunSpec (Tables 1–4), *k* is the number of genes from the input cluster in a given category and *f* is the total number of genes in a given category.

**Table 1.** Functional gene groups over-represented among genes whose expression in the W303 strain treated with cisplatin was higher than in the untreated strain

Category	*P*	In category from cluster	*k*	*f*
Methionine biosynthetic process [GO:0009086]	<1×10^−14^	*MET8 MET6 MET10 STR3 MET3 MET5 HOM6 MET14 MET1 MHT1 MET17 ADI1 MET2 MET22 MET16*	15	31
Cysteine biosynthetic process [GO:0019344]	3.786×10^−14^	*CYS3 MET10 CYS4 MET3 MET5 MET14 MET17 MET16*	8	12
Oxidation–reduction process [GO:0055114]	3.985×10^−7^	*MET8 AAD4 MXR1 AAD6 AAD16 MET10 FMO1 SER33 MET5 HOM6 MTD1 ADI1 ZWF1 MET16*	14	272
Cellular amino acid metabolic process [GO:0006520]	5.244×10^−7^	*CYS3 STR3 YHR112C SPS100 HOM6 MET17*	6	31
Amino acid transport [GO:0006865]	3.423×10^−6^	*AGP3 MUP1 MUP3 YCT1 MMP1 SAM3*	6	42
Sulfur compound metabolic process [GO:0006790]	3.395×10^−5^	*YHR112C OPT1 MET22*	3	7
Cellular aldehyde metabolic process [GO:0006081]	0.0001	*AAD4 AAD6 AAD16*	3	11
Transmembrane transport [GO:0055085]	0.0002	*SEO1 SUL1 AGP3 MUP1 VHT1 MUP3 OPT1 YCT1 MMP1 SUL2 SAM3*	11	303
*S*-adenosylmethionine biosynthesis [GO:0006556]	0.0003	*SAM2 SAM1*	2	3
Glycine catabolic process [GO:0006546]	0.0003	*GCV3 GCV1*	2	3
Glycine metabolic process [GO:0006544]	0.0006	*GCV1 SHM2*	2	4
Purine nucleotide biosynthetic process [GO:0006164]	0.0007	*ADE1 MTD1 ADE17*	3	18
Sulfate transport [GO:0008272]	0.001	*SUL1 SUL2*	2	5
Folic acid-containing compound biosynthesis [GO:0009396]	0.003	*MTD1 FOL1*	2	9
‘*De novo*’ IMP biosynthetic process [GO:0006189]	0.003	*ADE1 ADE17*	2	9

**Table 2.** Functional gene groups over-represented among genes whose expression in the W303 strain treated with cisplatin was lower than in the untreated strain

Category	*P*	In category from cluster	*k*	*f*
Translation [GO:0006412]	6.493×10^−13^	*RPS8A RPS9B RPS16B RPS17B RPS8B RPL24A RPL9A RPS26A RPL26B RPS0A RPL2B RPL16A RPL22A RPL6B RPS18B RPS1B RPL18B RPL18A RPS7A*	19	318
Maturation of SSU-rRNA from tricistronic rRNA transcript (SSU-rRNA, 5.8S rRNA, LSU-rRNA) [GO:0000462]	8.919×10^−5^	*RPS8A RPS9B RPS16B RPS8B RPS1B*	5	60
Ribosomal small subunit assembly [GO:0000028]	0.0001	*RPS17B NSR1 RPS0A*	3	14
Ammonium transport [GO:0015696]	0.0008	*ATO2 MEP3*	2	6
rRNA export from nucleus [GO:0006407]	0.0011	*RPS26A RPS0A RPS18B*	3	27
Gene conversion at mating-type locus, DNA double-strand break formation [GO:0000728]	0.0077	*HO*	1	1
Cytidine transport [GO:0015861]	0.0077	*FCY2*	1	1
Adenine catabolic process [GO:0006146]	0.0077	*AAH1*	1	1
Positive regulation of glycolysis [GO:0045821]	0.0077	*TYE7*	1	1
Tyrosine transport [GO:0015828]	0.0077	*TAT1*	1	1

**Table 3.** Functional gene groups over-represented among genes whose expression in the Δ*sky1* mutant strain was higher than in the W303 strain

Category	*P*	In category from cluster	*k*	*f*
Thiamine biosynthetic process [GO:0009228]	5.281×10^−5^	*THI13 THI5 THI11 THI12 THI20*	5	17
Fatty acid β-oxidation [GO:0006635]	0.0018	*POX1 POT1 TES1*	3	10
Cytochrome *c*-haem linkage [GO:0018063]	0.0020	*CYT2 CYC2*	2	3
NAD biosynthesis via nicotinamide riboside salvage pathway [GO:0034356]	0.0020	*URH1 PNP1*	2	3
Lipid metabolic process [GO:0006629]	0.0037	*YAT1 POX1 POT1 YJR107W IRS4 YSR3*	6	58
Response to stress [GO:0006950]	0.0059	*PAU8 ARO4 MRK1 DFM1 TOS3 PAU14 PAU1 DAN4 ICT1 FMP41*	10	152

**Table 4.** Functional gene groups over-represented among genes whose expression in the Δ*sky1* mutant strain was lower than in the W303 strain

Category	*P*	In category from cluster	*k*	*f*
rRNA processing [GO:0006364]	<1×10^−14^	*SPB1 NOP14 BFR2 FCF1 UTP6 SNM1 NOP16 NSA2 RAI1 NOP7 EFG1 RRP3 IMP3 MRT4 RRP14 EBP2 SOF1 FYV7 DIP2 NOP56 UTP21 UTP15 NOP2 DBP6 NOP58 MOT1 NAN1 BMS1 TIF6 MRD1*	30	195
Ribosome biogenesis [GO:0042254]	8.427×10^−14^	*SPB1 NOP14 FCF1 UTP6 NOP16 NSA2 NOP7 RRP3 IMP3 MRT4 RRP14 EBP2 SOF1 RIX7 DIP2 NOP56 RSA3 UTP21 UTP15 NOP2 DBP6 NOP8 NOC2 NOP58 NAN1 BMS1*	26	170
Dipeptide transport [GO:0042938]	0.0006	*DAL5 PTR2*	2	2
Cellular amino acid metabolic process [GO:0006520]	0.0010	*GDH3 CHA1 IRC7 SPS100 URA2*	5	31
Regulation of meiosis [GO:0040020]	0.0023	*RCK1 IME1 UME1*	3	11
Positive regulation of transcription from RNA polymerase I promoter [GO:0045943]	0.0030	*CTK1 UTP15 NAN1*	3	12
Glutamate biosynthetic process [GO:0006537]	0.0039	*GDH3 IDP1 GLT1*	3	13
Dephosphorylation [GO:0016311]	0.0085	*PHO11 PHO5 PHO12 SDP1*	4	32
rRNA methylation [GO:0031167]	0.0091	*TRM7 SPB1*	2	6

#### Analysis of expression by qRT (real-time)-PCR.

Total RNA isolated as described previously was converted into cDNA and labelled with the KAPA SYBR FAST universal one-step qRT-PCR kit (Kappa Biosystems). PCR primers for individual genes selected after the microarray analysis were designed with the Universal Probe Library Assay Design Center (https://www.roche-applied-science.com/sis/rtpcr/upl/index.jsp?id=uplct_030000) developed by Roche Diagnostics to generate 60–85 bp amplicons with a *T*_m_ of 59 or 60 °C. The list of primers is given in Table S9 (available in the online Supplementary Material). The ECO Real-Time PCR System was used for the experiments (Illumina) and calculations were made by the 2^–ΔΔ^*^C_t_^* method (Livak & Schmittgen, 2001). Three independent RNA extractions were assayed for each strain or condition. The mRNA levels of the selected genes were corrected by the geometric mean of the mRNA levels of *HHO1*, *TAF10* and *ALG9* – a selection of control genes which were verified previously to be constitutive in the assayed conditions and not affected by the Δ*sky1* deletion. A *t*-test was applied to evaluate the differences between Δ*C*_t_ values (*C*_t_ values normalized with reference genes) of control and treated samples with a *P* = 0.05.

#### Cellular accumulation of cisplatin and determination of sulfur content.

The yeast cells (W303 and derivatives, W303-Δ*sky1* and W303-Δ*seo1*) were pre-cultured overnight in 10 ml SD prepared as described above. The following day, the cells were inoculated at initial OD_600_ 0.4 in SD and grown at 30 °C and 250 r.p.m. When cells reached OD_600_ 0.6, the cultures from each strain were treated with cisplatin at a final concentration of 600 µM. The treatment was done at 30 °C and 250 r.p.m. during 4 h in darkness. Cells from 10 ml of each culture were harvested by centrifugation and washed 3 times with ice-cold PBS. The pellets were resuspended in 1 ml PBS. An aliquot of 0.1 ml cell suspension was utilized for the protein assay and the remainder was digested in 70 % nitric acid. Cell lysates were heated for 2 h at 65 °C, diluted to 5 % nitric acid, and assayed for platinum and sulfur content by means of high-resolution inductively coupled plasma MS. An aliquot of 10 ml of each culture was also used for DNA extraction as described by Hoffman & Winston (1987). The final pellet was resuspended in 0.5 ml PBS and the DNA concentration was measured. The samples were digested in 70 % nitric acid for 2 h at 65 °C, diluted to 5 % nitric acid and assayed for platinum as described above. Two biological replicates and two technical replicates were carried out. A *t*-test was applied to evaluate the differences between means. Statistically significant changes and their *P* values are indicated in Fig. 3.

#### Cytotoxicity assay.

Sensitivity to the cytotoxic effect of cisplatin was assessed using a colony formation assay. Cultures (1 ml SD) containing a total of 6×10^6^ cells were exposed for 4 h to cisplatin at concentrations of 0, 0.5, 1.0, 2.0, 2.5 and 5 mM, washed once in PBS, resuspended in YPD medium (2% glucose, 2% Bacto peptone, 1% yeast extract), diluted 1 : 4000, and plated onto YPD agar plates. After 2 days of growth at 30 °C, the number of colonies was counted. The IC_50_ was defined as the drug concentration that reduced the number of c.f.u. to 50 % of the value in a control culture not exposed to the drug. Each experiment was repeated three times with duplicate cultures for each drug concentration. A *t*-test was applied to evaluate the differences between means.

#### Glutathione determination.

The yeast strain W303 and its derivatives, W303-Δ*sky1* and W303-Δ*seo1* were treated with 600 µM cisplatin as described above. Aliquots of 50 ml of each culture were harvested and cells resuspended in 10 ml ice-cold PBS. Cells were washed twice with ice-cold PBS solution and resuspended in 350 µl PBS. Aliquots of 50 µl were reserved for protein determination (Bradford, 1976). To the rest, 300 µl 10 % sulfosalicylic acid was added. This cell suspension was mixed with 600 µl glass beads and cells were lysed by vortexing for 3 min, using 30 s pulses with intervals of 30 s on ice. The extracts were spun down in a microfuge at 4 °C to remove cell debris and protein precipitate. The supernatant was then diluted 1 : 5, and total glutathione and GSSG were determined using the 5,5′-dithio-bis(2-nitrobenzoic acid) (DTNB)-GSSG reductase recycling assay as described previously (Rhieu *et al.*, 2013). Each experiment was repeated three times with duplicate cultures for each drug concentration. A *t*-test was applied to evaluate the differences between means.

## Results

### Effect of cisplatin on the yeast transcriptome

The changes in mRNA levels produced by addition of 600 µM cisplatin in W303 and W303-Δ*sky1* cells grown in SD media were recorded, normalized and analysed for statistical significance as described in Methods. Only those with a ≥1.6-fold change and a *P*<0.01 according to the analysis with LIMMA (Smyth, 2005) were considered for further analysis. The original and normalized data from this study are freely available from the Gene Expression Omnibus database (http://www.ncbi.nlm.nih.gov/geo/info/linking.html); the accession number of the series is GSE41094. Processed data about the DEGs are available in Tables S1–S6). Selected genes were also analysed by qPCR to validate remarkable results as explained along the text.

There is a previous report of a transcriptome analysis describing the effect of cisplatin upon the yeast strain BY4730 (Caba *et al.*, 2005). However, although using the same drug concentration as in our study, the effects observed were limited to only five genes (*HUG1*, *ECM4* and YLR279W upregulated, and *HO* and *SCW11* downregulated). Here, we obtained a set of 122 differentially expressed genes: 69 upregulated (Table S1) and 53 downregulated (Table S2). The observed differences might be explained because the effect of cisplatin treatment is strain-dependent (Rodríguez Lombardero *et al.*, 2012), and also varies in relation to cell culture and treatment conditions, these being different in the two studies.

The functional distribution of DEGs was analysed with FunSpec as described in Methods. Analysing the effects of cisplatin treatment on W303 cells, functional groups over-represented among upregulated genes (Table 1) were those related to sulfate assimilation and metabolism of sulfur-containing compounds, e.g. sulfate transport (GO:0008272), transport of sulfur-containing amino acids (*AGP3*, *MUP1*, *MUP3*, *MMP1* and *SAM3*) or those necessary for methionine (GO:0009086), cysteine (GO:0019344) and *S*-adenosylmethionine (AdoMet) biosynthesis (GO:0006556). [Gene Ontology (GO) identifiers are given in parentheses.] Several genes related to purine nucleotide biosynthesis were also upregulated (*ADE1*, *ADE17* and *MTD1*) as well as others from glycine metabolism (*GCV3*, *GCV1* and *SMH2*) and folic acid biosynthesis (*FOL1*), as shown in Table 1.

Functional groups over-represented among downregulated genes (Table 2) were related to ribosome biogenesis, and included *RPS8A*, *RPS9B*, *RPS16B*, *RPS8B* and *RPS1B*, necessary for maturation of small subunit rRNA from tricistronic rRNA transcript, and *RPS17B*, *NSR1*, *RPS0A*, *RPS26A* and *RPS18B*, participating in ribosomal small subunit assembly and/or rRNA export from the nucleus. Among the other genes also downregulated by cisplatin treatment, but not directly related to ribosome biogenesis, were *TYE7* (positive regulator of glycolysis), *FCY2* (purine-cytosine permease), *AAH1* (adenine deaminase), *ATO2* and *MEP3* (ammonium transport), and *HO* (homothallic switching endonuclease, necessary for mating type switch). *FCY2* and *HO* have been related previously to the yeast response to cisplatin treatment in transcriptome and deletome studies (Caba *et al.*, 2005; Huang *et al.*, 2005).

### Effect of Sky1 depletion on the yeast transcriptome

There were 177 genes upregulated (Table S3) and 179 downregulated (Table S4) when *SKY1* was deleted. The functional distribution of upregulated genes analysed with FunSpec showed that most of them did not have a previously assigned function. Among the genes with known function (Table 3), there were groups of inter-related genes, which take part in the metabolism of lipids (GO:0006635 and 0006629), response to stress (GO:0006950), those related to cytochrome *c* haem linkage (GO:0018063), NAD^+^ biosynthesis via the nicotinamide riboside salvage pathway (GO:0034356) and thiamine biosynthetic process (GO:0009228).

The functional distribution of downregulated genes after *SKY1* deletion (Table 4) showed enriched groups, such as those participating in rRNA processing (GO:0006364), ribosome biogenesis (GO:0042254) and positive regulation of transcription from the RNA polymerase I promoter (GO:0045943). Three genes associated with glutamate biosynthesis (*GDH3*, *IDP1* and *GLT1*) and four genes related to dephosphorylation (*PHO11*, *PHO5*, *PHO12* and *SDP1*) were also downregulated, and included in Table 4. A selection of genes regulated by Sky1 in the DNA arrays was validated by qPCR (Table 5).

**Table 5.** Expression analysis by qPCR (*n* = 3)

Gene	Δ*sky1* NT/W303 NT	
	Fold change	*P*
*DIN7*	+2.30	0.049
*FOX2*	+3.04	0.016
*HUG1*	+2.06	0.001
*ICL1*	+2.79	0.038
*MHT1*	+3.23	0.018
*ODC2*	+1.77	0.049
*POT1*	+2.47	0.046
*POX1*	+2.14	0.025
*SPP382*	+2.28	0.047
CTK1*	−1.94	0.016
*ECM2*	−1.78	0.042
*GDH3*	−5.81	0.020
*GLT1*	−2.04	0.018
*HRP1*	−2.32	0.039
*IDP1*	−2.17	0.033

NT, not treated; +, upregulation; –, downregulation.

* *n* = 6.

### Effect of Sky1 depletion on the response of the yeast transcriptome to cisplatin treatment

**Fig. 1.**
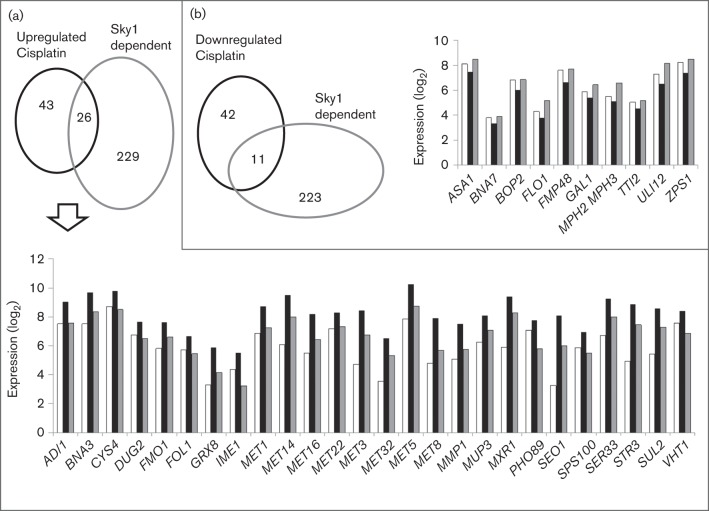
Genes that change their mRNA levels in response to cisplatin treatment and are dependent on Sky1 function. (a) Upregulated genes. The Venn diagram shows the intersections between genes in Tables S1 and S6. (b) Downregulated genes. The Venn diagram shows the intersections between genes in Tables S2 and S5. Histograms show the levels of gene expression corresponding to the genes from intersections of the accompanying Venn diagrams in the three conditions compared: white bars, W303 strain; black bars, W303 strain treated with cisplatin; grey bars, Δ*sky1* strain treated with cisplatin.

**Fig. 2.**
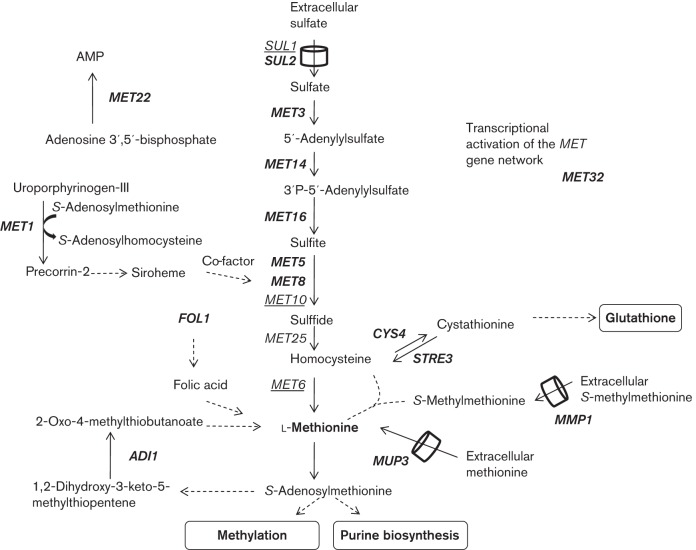
Sulfur uptake and sulfur assimilation pathways in *S. cerevisiae*. Genes upregulated by cisplatin treatment and that are Sky1 dependent are in bold type. Those upregulated by cisplatin but not dependent on Sky1 function are underlined. The cylinder symbols represent membrane transporters.

In order to understand the role of Sky1 in the response of the yeast transcriptome to cisplatin, we compared the list of DEGs shown in Tables S1 (genes upregulated by cisplatin in the W303 strain) and S6 (genes downregulated in the W303-Δ*sky1* treated strain), as well as those from Tables S2 (genes downregulated by cisplatin in the W303 strain) and S5 (genes upregulated in the W303-Δ*sky1* treated strain). The analysis is represented in the Venn diagrams in Fig. 1, and the functions of the genes found in the intersections are included as Tables S7 and S8. Levels of relative expression of these genes are also shown in Fig. 1. A new issue revealed by this analysis was that the cisplatin-induced upregulation of genes related to the metabolism of sulfur-containing compounds (*MET1*, *MET14*, *MET16*, *MET22*, *MET3*, *MET32*, *MET5*, *MET8*, *STRE3* and *SUL2*) was partially dependent on Sky1 function (Fig. 1a, Table S7). Fig. 2 summarizes the metabolic pathways associated with these genes. The expression pattern of selection of genes depicted in Fig. 2 was validated by qPCR and the results (Table 6) confirmed that the increase caused by cisplatin was reversed to a decrease in expression in the comparison of W303 versus W303-Δ*sky1* treated cells.

**Table 6.** Expression analysis by qPCR (*n* = 3)

Gene	W303 T/W303 NT		Δ*sky1* T/W303 T	
	Fold change	*P*	Fold change	*P*
*MET1*	+4.08	0.008	−7.13	0.031
*MET14*	+18.26	<0.001	−2.35	0.025
*MET16*	+6.24	0.003	−2.40	0.003
*MET32*	+4.60	<0.001	−2.19	<0.001
*MET5*	+5.24	0.009	−3.07	<0.001
*MET8*	+1.82	0.034	−1.79	0.025
*STR3*	+13.14	<0.001	−4.14	0.074
*SUL2*	+10.23	0.003	−2.31	0.007
*SEO1*	+5.77	<0.001	−3.31	0.042

T, treated with cisplatin; NT, not treated; +, upregulation; –, downregulation.

The copper transporter Ctr1 has been reported to import cisplatin into yeast cells (Ishida *et al.*, 2002). In the analysis of data from the DNA arrays, we also focused on changes produced upon expression of permease genes that could be related to cisplatin transport. In the transcriptome analysis, the gene encoding a permease and with the highest transcriptional response to cisplatin was *SEO1*. The changes observed with the DNA arrays in the expression of *SEO1* in response to cisplatin (upregulation) and the consequence of *SKY1* deletion in this response (downregulation) were also confirmed by qPCR (Table 6). The actual substrates transported by Seo1 have not yet been described, although it has been suggested that it might be involved in the transport of sulfur compounds (Isnard *et al.*, 1996).

In addition, 11 genes were downregulated in response to cisplatin in a Sky1-dependent manner (Fig. 1b, Table S8). As shown in Table S8, two of these genes encoded subunits of the ASTRA complex and were related to chromatin remodelling (*ASA1* and *TTI2*).

### Intracellular platinum and sulfur content in the W303 strain and its derivatives, W303-Δ*sky1* and W303-Δ*seo1*

**Fig. 3.**
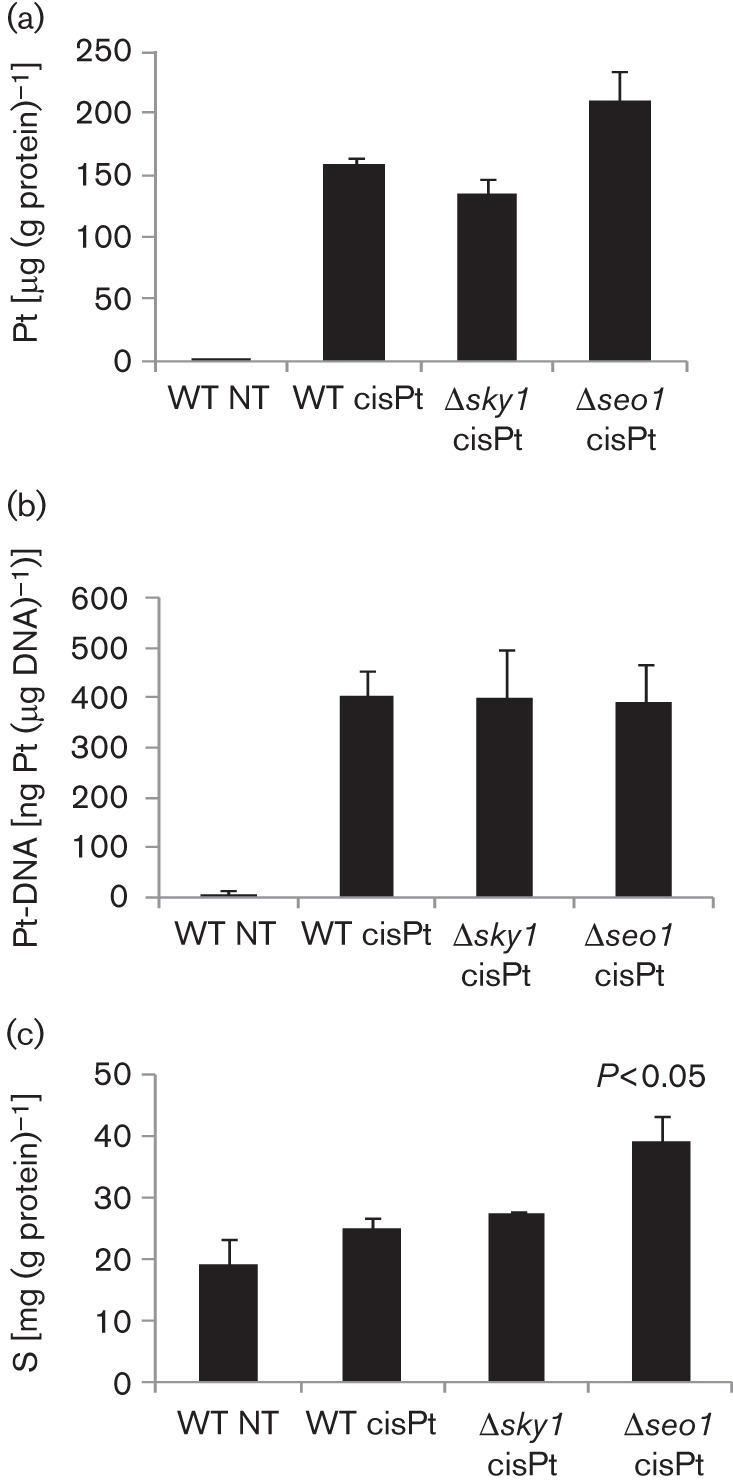
Platinum and sulfur content in W303, W303-Δ*sky1* and W303-Δ*seo1* strains after cisplatin treatment. (a) Platinum content, (b) platinum in genomic DNA and (c) sulfur content. NT, not treated; cisPt, cisplatin treatment. Comparisons were made with W303 treated cells. The *P* value is indicated when significant differences were found.

The yeast strain W303 and derivatives, W303-Δ*sky1* W303-Δ*seo1*, were treated with 600 µM cisplatin, and intracellular levels of platinum and sulfur were compared with those measured in W303 untreated cells. The levels of platinum incorporation into genomic DNA were also determined. The results (Fig. 3) showed that the content of intracellular platinum after cisplatin treatment did not change significantly in the Δ*sky1* or Δ*seo1* deletants, as compared with the W303 strain (Fig. 3a). According to these results and a previous report (Schenk *et al.*, 2002), resistance to cisplatin in Δ*sky1* cells was probably not produced by decreased intracellular drug concentration. DNA platination was not affected either (Fig. 3b), which indicated that the differences in resistance between the strains were due to signals generated after the formation of DNA adducts. In the W303 and W303-Δ*sky1* strains, the content of intracellular sulfur did not increase after cisplatin treatment, but in the Δ*seo1* deletant there was a significant increase after cisplatin treatment compared with the W303 treated strain (Fig. 3c).

### Effects of downregulation of the methionine pathway and *SEO1* deletion on cisplatin resistance

**Fig. 4.**
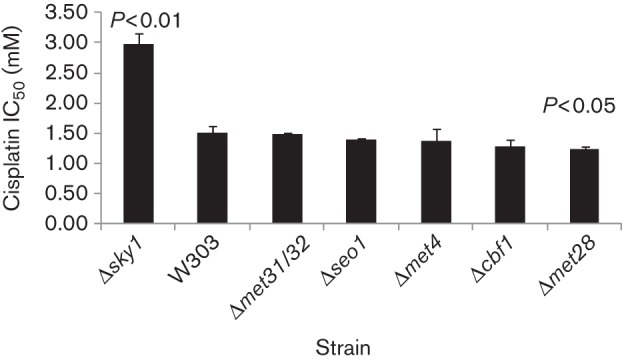
IC_50_ calculated from cytotoxicity assays. Comparisons were done versus W303 treated cells. The *P* value is indicated when significant differences were found.

As reported above for the W303 strain, cisplatin treatment increased the mRNA levels of a series of genes related to sulfur assimilation and the metabolism of sulfur compounds (Table 1) in a Sky1-dependent manner (Figs 1 and 2). Considering that the W303-Δ*sky1* strain is more resistant to cisplatin than W303, we also tested if the deletion of genes encoding transcriptional regulators of the methionine pathway (*MET4*, *MET*28, the two homologues *MET31/MET32* or *CBF1*) (Lee *et al.*, 2010), as well as the deletion of *SEO1*, produced changes in the sensitivity to cisplatin. The cytotoxic assays were performed with the selected strains as reported in Methods and the IC_50_ was calculated. Results (Fig. 4) confirmed the increased resistance of a Δ*sky1* strain as reported previously (Huang *et al.*, 2005). No significant changes were observed for the other strains analysed, with the exception of Δ*met28*, which was more sensitive to cisplatin than the WT.

### Intracellular glutathione levels after cisplatin treatment

**Fig. 5.**
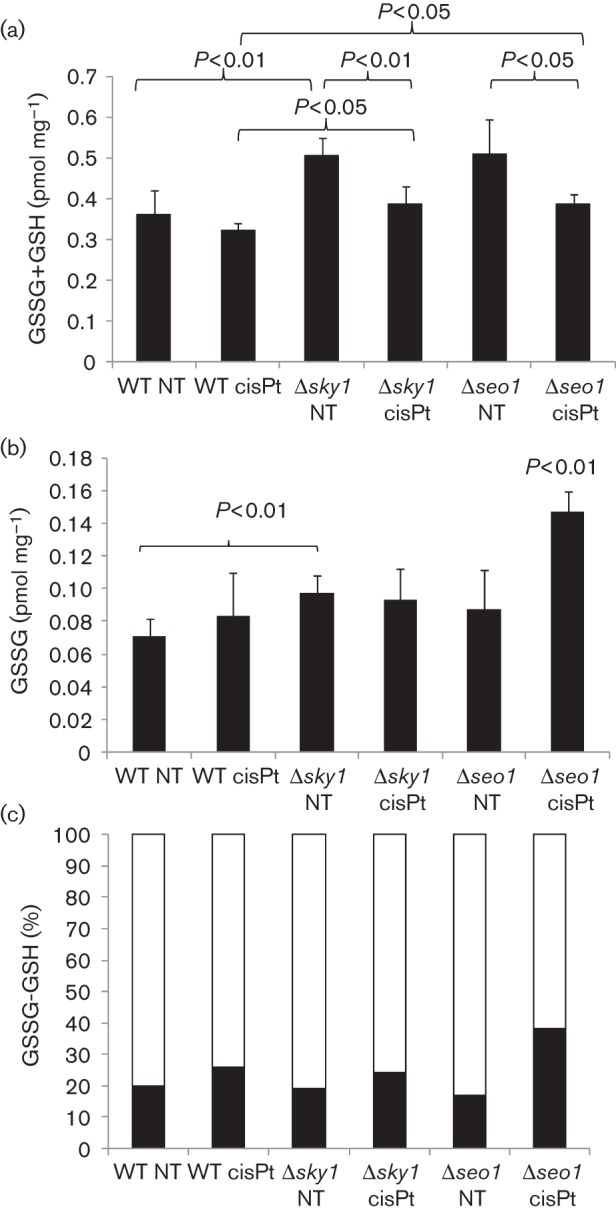
Glutathione determination in W303, W303-Δ*sky1* and W303-Δ*seo1* strains after cisplatin treatment (a) Total (GSSG+GSH), (b) GSSG, and (c) per cent GSSG (black) and GSH (white). NT, not treated; cisPt, cisplatin treatment. The *P* value is indicated when statistically significant differences were found and the corresponding pair comparisons are indicated by brackets. In (b), the absence of brackets in the comparison of W303*-*Δ*seo1* cisPt indicates that all pair comparisons are significant.

Levels of glutathione were determined in the W303, W303-Δ*sky1* and W303-Δ*seo1* strains as described in Methods. The measured total intracellular glutathione concentration (GSSG+GSH) was in the range of previous determinations in yeast cells (Rhieu *et al.*, 2013). In the absence of cisplatin, the total glutathione content was increased in both mutants as compared with the WT strain (Fig. 5a). Differences in the total glutathione content between treated and untreated cells were not statistically significant in the WT, but significant decreases were found in the mutants (Fig. 5a). In the absence of treatment, values of GSSG were only higher in the W303-Δ*sky1* strain than in W303 (Fig. 5b). For the W303-Δ*seo1* treated strain, the comparisons with all the other strains and conditions differed significantly in GSSG content (Fig. 5b). With regard to the GSSG/GSH ratio after cisplatin treatment, the major increase was observed in the W303-Δ*seo1* strain (Fig. 5c). Cisplatin treatment has been related to oxidative stress (Martins *et al.*, 2008; Pratibha *et al.*, 2006) and changes in the expression of genes encoding enzymes related to oxidation–reduction processes were observed in the yeast strains analysed in this study after cisplatin treatment (Table 1). However, a comparison of the GSSG/GSH ratios in the W303 and W303*-*Δ*sky1* strains could not explain the higher resistance to cisplatin observed in the null mutant by higher levels of available GSH (Fig. 5c).

## Discussion

### Sulfur metabolism and transcriptional response to cisplatin treatment

The combination of deletome and transcriptome analyses is useful to identify regulatory processes in response to external stimuli (Jin *et al.*, 2008). A comparison of genes whose expression changed in response to cisplatin in our study (Tables S1 and S2) and those whose deletions changed the sensitivity or resistance of the strain to the drug (Burger *et al.*, 2000; Huang *et al.*, 2005; Wu *et al.*, 2004) confirms that there is no direct correlation between transcriptome and deletome analyses as proposed previously (Birrell *et al.*, 2002; Jin *et al.*, 2008). For instance, several genes related to DNA repair mechanisms, such as *RAD* or *REV* genes and others, whose deletions increase cisplatin sensitivity in yeast (Burger *et al.*, 2000; Wu *et al.*, 2004), do not change their mRNA levels after cisplatin treatment in our study. This is consistent with the assumption that many other post-transcriptional events are part of the regulatory responses elicited by cisplatin, which are not detectable in the transcriptome analyses. Nevertheless, our transcriptome approach has identified genes from sulfate assimilation and sulfur metabolism as targets of cisplatin treatment in a response partially dependent on *SKY1* function (Tables 1 and 6, Figs 1 and 2), which have not been identified in previous deletome analyses. The reduction of sulfate to sulfide through the sulfate assimilation pathway allows the synthesis of homocysteine. This metabolite is a precursor of methionine and AdoMet through the methyl cycle, and also of cysteine and glutathione through the *trans*-sulfuration pathway. In turn, AdoMet is required in the biosynthesis of sirohaem, which is necessary for sulfate assimilation. Fig. 2 shows the effects of cisplatin and *SKY1* deletion on these pathways as deduced from the transcriptome analysis. As indicated in Fig. 2, increased transcription of *MMP1* and *MUP3* genes, necessary for the incorporation of methionine or *S*-methylmethionine from the medium, might also play an important role in the yeast response to cisplatin.

As *Met4* is the transcriptional activator of the genes from the assimilation of sulfate and sulfur compound biosynthesis, we also tested if deletion of *MET4* or the genes encoding other regulatory proteins (*MET31*, *MET32*, *CBF1* and *MET28*), which lack intrinsic transcriptional activation and act as adaptors for recruiting Met4 to appropriate promoters (Blaiseau *et al.*, 1997; Kuras *et al.*, 1996), caused a decrease in cisplatin IC_50_ in cytotoxicity assays (Fig. 4). No significant change was observed in most of the deleted strains, thus indicating that the cellular response to cisplatin does not depend on a mechanism related to the transcription factor Met4. We have only found a small, although statistically significant change, from 1.50 to 1.23 mM, in the Δ*met28* deletant. It has been proposed that Met4-activated transcription necessarily depends on Met31 and Met32, whereas the dependency on Cbf1 or Met28 selects distinct sulfur metabolic processes (Lee *et al.*, 2010), separating the assimilation of inorganic sulfate (Cbf1) from the synthesis of organic compounds (Met28). Therefore, sensitivity to cisplatin might be affected by dysregulation of the synthesis of specific sulfur organic compounds, rather than by a general increase in all the metabolic pathways downstream of sulfate assimilation.

Although cisplatin elicits a wide transcriptional response affecting genes from the assimilation and metabolism of sulfur compounds, no significant change in total intracellular sulfur content after cisplatin treatment was observed in W303 (Fig. 3c), which might be explained if increased synthesis of sulfur compounds is accompanied by an active export of one or more of them to the medium. One of the genes related to the transport of sulfur compounds and with a high transcriptional response to cisplatin treatment is *SEO1.* However, the substrate of this permease has not yet been identified (Isnard *et al.*, 1996). The expression of *SEO1* in response to cisplatin partially depends on Sky1 (Fig. 1a, Table 6). We have found that the deletion of *SEO1* significantly increases intracellular levels of sulfur (Fig. 3c) without affecting platinum levels or DNA platination (Fig. 3a, b). This argues in favour of its participation in the export of sulfur compounds, but not in complex with platinum compounds.

The fact that a cisplatin-induced transcriptional response upregulates the expression of genes related to sulfate assimilation raises the question of the metabolic use of homocysteine for the biosynthesis of glutathione via cystathionine or for the biosynthesis of l-methionine and AdoMet. Cisplatin exhibits a high affinity toward sulfur donors, such as cysteine, methionine or glutathione. Thus, the formation of stable platinum–sulfur bonds with them might contribute to the mechanism of detoxification and resistance. In mammalian cells, data are still controversial with the assumption that glutathione functions in cisplatin chelation and detoxification. As favourable arguments, depletion of glutathione increased sensitization to cisplatin toxicity in osteosarcoma cells (Komiya *et al.*, 1998). In addition, high resistance to cisplatin in human ovarian cancer cell lines was associated with a marked increase of glutathione synthesis (Godwin *et al.*, 1992), and reactions of *cis*- and *trans*-[PtCl_2_(NH_3_)_2_] with reduced glutathione (GSH) inside intact red blood cells have been reported (Berners-Price & Kuchel, 1990). In contrast, in other studies there was no correlation between cellular resistance and glutathione levels (Boubakari *et al.*, 2004; Bracht *et al.*, 2006). To the best of our knowledge, none of these mechanisms have previously been studied in yeast. We have thus measured the glutathione content in response to cisplatin treatment and observed that there is no increase in total glutathione content after treatment of the W303 strain (Fig. 5a). Accordingly, expression of the genes involved in the biosynthesis of glutathione from cysteine (*GSH1* and *GSH2*) is not enhanced in the transcritome data. In the Δ*sky1* mutant, the decrease in utilization of homocysteine for glutathione biosynthesis, as suggested by the significant decrease of total glutathione after cisplatin treatment (Fig. 5a), would allow the higher production of cysteine and methionine. This could be advantageous for resistance against cisplatin if, as recently reported, cysteine and methionine are efficient chelants for cisplatin (Zimmermann *et al.*, 2005), whilst major glutathione cisplatin adducts, [Pt(SG)_2_], can only account for ≤20 % of the total cisplatin adducts formed inside the cell (Kasherman *et al.*, 2009).

If homocysteine is not employed for glutathione synthesis as explained above, it can be expected to be channelled into the biosynthesis of methionine and AdoMet as well (Fig. 2). Thus, in the cellular response to cisplatin it is also possible that AdoMet, derived from methionine, could be used in the biosynthesis of purine nucleotides needed for DNA repair. This view is supported by the transcriptional data reported here. Several genes related to purine nucleotide biosynthesis (*ADE1*, *ADE17* and *MTD1*) as well as others from glycine metabolism (*GCV3*, *GCV1* and *SMH2*) and folic acid biosynthesis (*FOL1*), previously found to be co-regulated with the synthesis of enzymes of the purine biosynthetic pathway (Denis & Daignan-Fornier, 1998), are upregulated after cisplatin treatment (Table 1). This stimulation of purine biosynthesis pathways, as well as that of metabolites consumed during *de novo* purine biosynthesis like glycine and 10-formyl tetrahydrofolate, could provide the necessary nucleotides for DNA repair mechanisms, which are well known to participate in the response to cisplatin (Basu & Krishnamurthy, 2010). Also supporting our results, transcriptome and metabolome analyses in cancerous cell lines demonstrated a relation between cisplatin sensitivity and nucleotide synthesis by both salvage and *de novo* pathways (Cavill *et al.*, 2011).

### Cisplatin treatment and stress response

Sulfur metabolism is involved in multiple facets of cellular metabolism related to several responses to stress. Indeed, Met4-dependent transcriptional activation of genes related to assimilation and metabolism of sulfur compounds has been reported previously in response to cadmium and other metal-induced stress (Fauchon *et al.*, 2002; Jin *et al.*, 2008). Yeast cells exposed to cadmium adapt to the extreme requirement of sulfur for GSH synthesis by sulfur sparing. Selective translation of proteins with a low content of sulfur, including enzymes of the sulfur metabolic pathway, allows the sparing of sulfur-containing amino acids (Fauchon *et al.*, 2002). The observed transcriptional activation of genes related to assimilation and metabolism of sulfur compounds after cisplatin treatment differs from metal-induced stress (Fauchon *et al.*, 2002), since it is not accompanied by an increase in glutathione levels (Fig. 5).

Activation of genes from sulfur metabolism in response to metals (Fauchon *et al.*, 2002; Jin *et al.*, 2008) or after Met4-induced hyper-activation (Lee *et al.*, 2010) has been co-associated with downregulation of ribosome biogenesis genes. This co-association is also observed in our study. The decrease in cell proliferation that usually accompanies the stress might cause the observed downregulation of genes related to ribosome synthesis. We have found that cisplatin treatment and Sky1 depletion (Tables 2 and 4, respectively) cause downregulation of genes related to rRNA processing and ribosome biogenesis. In support of this result, cisplatin inhibits rRNA synthesis in HeLa cell cultures by a mechanism that affects initiation of transcription by RNA polymerase I (Jordan & Carmo-Fonseca, 1998). An interesting result from our study is that in the Δ*sky1* strain (Table 4) there is a downregulation of three genes (*CTK1*, *UTP15* and *NAN1*) which encode proteins that cause a positive regulation of transcription from the RNA polymerase I promoter (Table S8). The Sky1-dependent regulation of *CTK1* has been verified and confirmed by qPCR (Table 5).

## Conclusions

The transcriptional response to cisplatin and its dependence on Sky1 function has been evaluated. There is a high increase in mRNA levels of genes related to sulfate assimilation and metabolism of sulfur compounds upon cisplatin treatment, which depends partially on Sky1 function. *SEO1* expression increases in this response and its deletion increases the intracellular sulfur content.

Several putative mechanisms may explain the increased cisplatin resistance of the W303-Δ*sky1* strain when considering the data on the platinum, sulfur and glutathione content, and transcriptional regulation of sulfur metabolism. Thus, resistance does not depend on a decrease in the intracellular levels of cisplatin, DNA platination or variations in the GSH/GSSG ratio. Regulated expression of genes from sulfur metabolism, together with the observed decrease of intracellular glutathione levels in the Δ*sky1* strain, whilst maintaining the total sulfur content, suggests the preferential use of homocysteine for cysteine and methionine production. The formation of cisplatin complexes with these thiols could be envisaged as a mechanism of increased resistance.

## References

[r1] Basu A. Krishnamurthy S. **(**2010**).** Cellular responses to cisplatin-induced DNA damage. J Nucleic Acids 2010, 1–16. 10.4061/2010/201367 20811617 PMC2929606

[r2] Benjamini Y. Hochberg Y. **(**1995**).** Controlling the false discovery rate: a practical and powerful approach to multiple testing. J R Stat Soc Series B Stat Methodol 57, 289–300.

[r3] Berners-Price S. J. Kuchel P. W. **(**1990**).** Reaction of *cis*- and *trans*-[PtCl_2_(NH_3_)_2_] with reduced glutathione inside human red blood cells, studied by ^1^H and ^15^N-{^1^H} DEPT NMR. J Inorg Biochem 38, 327–345. 10.1016/0162-0134(90)80006-J 2332767

[r4] Birrell G. W. Brown J. A. Wu H. I. Giaever G. Chu A. M. Davis R. W. Brown J. M. **(**2002**).** Transcriptional response of *Saccharomyces cerevisiae* to DNA-damaging agents does not identify the genes that protect against these agents. Proc Natl Acad Sci U S A 99, 8778–8783. 10.1073/pnas.132275199 12077312 PMC124375

[r5] Blaiseau P. L. Isnard A. D. Surdin-Kerjan Y. Thomas D. **(**1997**).** Met31p and Met32p, two related zinc finger proteins, are involved in transcriptional regulation of yeast sulfur amino acid metabolism. Mol Cell Biol 17, 3640–3648. 9199298 10.1128/mcb.17.7.3640 PMC232216

[r6] Boubakari B. K. Bracht K. Neumann C. Grünert R. Bednarski P. J. **(**2004**).** No correlation between GSH levels in human cancer cell lines and the cell growth inhibitory activities of platinum diamine complexes. Arch Pharm (Weinheim) 337, 668–671. 10.1002/ardp.200400620 15614830

[r7] Bracht K. Boubakari Grünert R. Bednarski P. J. **(**2006**).** Correlations between the activities of 19 anti-tumor agents and the intracellular glutathione concentrations in a panel of 14 human cancer cell lines: comparisons with the National Cancer Institute data. Anticancer Drugs 17, 41–51. 10.1097/01.cad.0000190280.60005.05 16317289

[r8] Bradford M. M. **(**1976**).** A rapid and sensitive method for the quantitation of microgram quantities of protein utilizing the principle of protein-dye binding. Anal Biochem 72, 248–254. 10.1016/0003-2697(76)90527-3 942051

[r9] Burger H. Capello A. Schenk P. W. Stoter G. Brouwer J. Nooter K. **(**2000**).** A genome-wide screening in *Saccharomyces cerevisiae* for genes that confer resistance to the anticancer agent cisplatin. Biochem Biophys Res Commun 269, 767–774. 10.1006/bbrc.2000.2361 10720490

[r10] Caba E. Dickinson D. A. Warnes G. R. Aubrecht J. **(**2005**).** Differentiating mechanisms of toxicity using global gene expression analysis in *Saccharomyces cerevisiae*. Mutat Res 575, 34–46. 10.1016/j.mrfmmm.2005.02.005 15878181

[r11] Cavill R. Kamburov A. Ellis J. K. Athersuch T. J. Blagrove M. S. Herwig R. Ebbels T. M. Keun H. C. **(**2011**).** Consensus-phenotype integration of transcriptomic and metabolomic data implies a role for metabolism in the chemosensitivity of tumour cells. PLoS Comput Biol 7, e1001113. 10.1371/journal.pcbi.1001113 21483477 PMC3068923

[r12] Dagher S. F. Fu X. D. **(**2001**).** Evidence for a role of Sky1p-mediated phosphorylation in 3′ splice site recognition involving both Prp8 and Prp17/Slu4. RNA 7, 1284–1297. 10.1017/S1355838201016077 11565750 PMC1370172

[r13] Denis V. Daignan-Fornier B. **(**1998**).** Synthesis of glutamine, glycine and 10-formyl tetrahydrofolate is coregulated with purine biosynthesis in *Saccharomyces cerevisiae*. Mol Gen Genet 259, 246–255. 10.1007/s004380050810 9749667

[r14] Fauchon M. Lagniel G. Aude J. C. Lombardía L. Soularue P. Petat C. Marguerie G. Sentenac A. Werner M. Labarre J. **(**2002**).** Sulfur sparing in the yeast proteome in response to sulfur demand. Mol Cell 9, 713–723. 10.1016/S1097-2765(02)00500-2 11983164

[r15] Fuertes M. A. Castilla J. Alonso C. Pérez J. M. **(**2003**).** Cisplatin biochemical mechanism of action: from cytotoxicity to induction of cell death through interconnections between apoptotic and necrotic pathways. Curr Med Chem 10, 257–266. 10.2174/0929867033368484 12570712

[r16] Gietz R. D. Akio S. **(**1988**).** New yeast–*Escherichia coli* shuttle vectors constructed with *in vitro* mutagenized yeast genes lacking six-base pair restriction sites. Gene 74, 527–534. 10.1016/0378-1119(88)90185-0 3073106

[r17] Gilbert W. Siebel C. W. Guthrie C. **(**2001**).** Phosphorylation by Sky1p promotes Npl3p shuttling and mRNA dissociation. RNA 7, 302–313. 10.1017/S1355838201002369 11233987 PMC1370088

[r18] Godwin A. K. Meister A. O’Dwyer P. J. Huang C. S. Hamilton T. C. Anderson M. E. **(**1992**).** High resistance to cisplatin in human ovarian cancer cell lines is associated with marked increase of glutathione synthesis. Proc Natl Acad Sci U S A 89, 3070–3074. 10.1073/pnas.89.7.3070 1348364 PMC48805

[r19] Hoffman C. S. Winston F. **(**1987**).** A ten-minute DNA preparation from yeast efficiently releases autonomous plasmids for transformation of *Escherichia coli*. Gene 57, 267–272. 10.1016/0378-1119(87)90131-4 3319781

[r20] Huang R. Y. Eddy M. Vujcic M. Kowalski D. **(**2005**).** Genome-wide screen identifies genes whose inactivation confer resistance to cisplatin in *Saccharomyces cerevisiae*. Cancer Res 65, 5890–5897. 10.1158/0008-5472.CAN-04-4093 15994967

[r21] Ishida S. Lee J. Thiele D. J. Herskowitz I. **(**2002**).** Uptake of the anticancer drug cisplatin mediated by the copper transporter Ctr1 in yeast and mammals. Proc Natl Acad Sci U S A 99, 14298–14302. 10.1073/pnas.162491399 12370430 PMC137878

[r22] Isnard A. D. Thomas D. Surdin-Kerjan Y. **(**1996**).** The study of methionine uptake in *Saccharomyces cerevisiae* reveals a new family of amino acid permeases. J Mol Biol 262, 473–484. 10.1006/jmbi.1996.0529 8893857

[r23] Jin Y. H. Dunlap P. E. McBride S. J. Al-Refai H. Bushel P. R. Freedman J. H. **(**2008**).** Global transcriptome and deletome profiles of yeast exposed to transition metals. PLoS Genet 4, e1000053. 10.1371/journal.pgen.1000053 18437200 PMC2278374

[r24] Jordan P. Carmo-Fonseca M. **(**1998**).** Cisplatin inhibits synthesis of ribosomal RNA *in vivo*. Nucleic Acids Res 26, 2831–2836. 10.1093/nar/26.12.2831 9611224 PMC147654

[r25] Kasherman Y. Sturup S. Gibson D. **(**2009**).** Is glutathione the major cellular target of cisplatin? A study of the interactions of cisplatin with cancer cell extracts. J Med Chem 52, 4319–4328. 10.1021/jm900138u 19537717

[r26] Kim H. K. Choi I. J. Kim C. G. Kim H. S. Oshima A. Michalowski A. Green J. E. **(**2011**).** A gene expression signature of acquired chemoresistance to cisplatin and fluorouracil combination chemotherapy in gastric cancer patients. PLoS ONE 6, e16694. 10.1371/journal.pone.0016694 21364753 PMC3041770

[r27] Komiya S. Gobhardt M. C. Mangham D. C. Inoue A. **(**1998**).** Role of glutathione in cisplatin resistance in osteosarcoma cell lines. J Orthop Res 16, 15–22. 10.1002/jor.1100160104 9565068

[r28] Kuras L. Cherest H. Surdin-Kerjan Y. Thomas D. **(**1996**).** A heteromeric complex containing the centromere binding factor 1 and two basic leucine zipper factors, Met4 and Met28, mediates the transcription activation of yeast sulfur metabolism. EMBO J 15, 2519–2529. 8665859 PMC450184

[r29] Lee T. A. Jorgensen P. Bognar A. L. Peyraud C. Thomas D. Tyers M. **(**2010**).** Dissection of combinatorial control by the Met4 transcriptional complex. Mol Biol Cell 21, 456–469. 10.1091/mbc.E09-05-0420 19940020 PMC2814790

[r30] Livak K. J. Schmittgen T. D. **(**2001**).** Analysis of relative gene expression data using real-time quantitative PCR and the 2^–ΔΔ^*^C^*T method. Methods 25, 402–408. 10.1006/meth.2001.1262 11846609

[r31] Martins N. M. Santos N. A. Curti C. Bianchi M. L. Santos A. C. **(**2008**).** Cisplatin induces mitochondrial oxidative stress with resultant energetic metabolism impairment, membrane rigidification and apoptosis in rat liver. J Appl Toxicol 28, 337–344. 10.1002/jat.1284 17604343

[r32] Medina I. Carbonell J. Pulido L. Madeira S. C. Goetz S. Conesa A. Tárraga J. Pascual-Montano A. Nogales-Cadenas R. **& other authors (**2010**).** Babelomics: an integrative platform for the analysis of transcriptomics, proteomics and genomic data with advanced functional profiling. Nucleic Acids Res 38 (Web Server), W210–W213. 10.1093/nar/gkq388 20478823 PMC2896184

[r33] Pérez R. P. **(**1998**).** Cellular and molecular determinants of cisplatin resistance. Eur J Cancer 34, 1535–1542. 10.1016/S0959-8049(98)00227-5 9893624

[r34] Pratibha R. Sameer R. Rataboli P. V. Bhiwgade D. A. Dhume C. Y. **(**2006**).** Enzymatic studies of cisplatin induced oxidative stress in hepatic tissue of rats. Eur J Pharmacol 532, 290–293. 10.1016/j.ejphar.2006.01.007 16458885

[r35] Rhieu S. Y. Urbas A. A. Lippa K. A. Reipa V. **(**2013**).** Quantitative measurements of glutathione in yeast cell lysate using ^1^H NMR. Anal Bioanal Chem 405, 4963–4968. 10.1007/s00216-013-6858-5 23471371

[r36] Robinson M. D. Grigull J. Mohammad N. Hughes T. R. **(**2002**).** FunSpec: a web-based cluster interpreter for yeast. BMC Bioinformatics 3, 35. 10.1186/1471-2105-3-35 12431279 PMC139976

[r37] Rodríguez Lombardero S. Vizoso Vázquez A. Rodríguez Belmonte E. González Siso M. I. Cerdán M. E. **(**2012**).** *SKY1* and *IXR1* interactions, their effects on cisplatin and spermine resistance in *Saccharomyces cerevisiae*. Can J Microbiol 58, 184–188. 10.1139/w11-124 22260231

[r38] Schenk P. W. Boersma A. W. Brandsma J. A. den Dulk H. Burger H. Stoter G. Brouwer J. Nooter K. **(**2001**).** *SKY1* is involved in cisplatin-induced cell kill in *Saccharomyces cerevisiae*, and inactivation of its human homologue, *SRPK1*, induces cisplatin resistance in a human ovarian carcinoma cell line. Cancer Res 61, 6982–6986. 11585720

[r39] Schenk P. W. Boersma A. W. Brok M. Burger H. Stoter G. Nooter K. **(**2002**).** Inactivation of the *Saccharomyces cerevisiae SKY1* gene induces a specific modification of the yeast anticancer drug sensitivity profile accompanied by a mutator phenotype. Mol Pharmacol 61, 659–666. 10.1124/mol.61.3.659 11854447

[r40] Shen H. Green M. R. **(**2006**).** RS domains contact splicing signals and promote splicing by a common mechanism in yeast through humans. Genes Dev 20, 1755–1765. 10.1101/gad.1422106 16766678 PMC1522072

[r41] Smyth G. K. **(**2005**).** LIMMA: linear models for microarray data. In Bioinformatics and Computational Biology Solutions using R and Bioconductor, pp. 397–420. Edited by Gentleman R. Carey V. Dudoit S. Irizarry R. Huber W. New York: Springer 10.1007/0-387-29362-0_23

[r42] Tizón B. Rodríguez-Torres A. M. Cerdán M. E. **(**1999**).** Disruption of six novel *Saccharomyces cerevisiae* genes reveals that YGL129c is necessary for growth in non-fermentable carbon sources, YGL128c for growth at low or high temperatures and YGL125w is implicated in the biosynthesis of methionine. Yeast 15, 145–154. 10.1002/(SICI)1097-0061(19990130)15:2<145::AID-YEA346>3.0.CO;2-J 10029993

[r43] Wan Y. W. Sabbagh E. Raese R. Qian Y. Luo D. Denvir J. Vallyathan V. Castranova V. Guo N. L. **(**2010**).** Hybrid models identified a 12-gene signature for lung cancer prognosis and chemoresponse prediction. PLoS ONE 5, e12222. 10.1371/journal.pone.0012222 20808922 PMC2923187

[r44] Windgassen M. Krebber H. **(**2003**).** Identification of Gbp2 as a novel poly(A)^+^ RNA-binding protein involved in the cytoplasmic delivery of messenger RNAs in yeast. EMBO Rep 4, 278–283. 10.1038/sj.embor.embor763 12634846 PMC1315891

[r45] Windgassen M. Sturm D. Cajigas I. J. González C. I. Seedorf M. Bastians H. Krebber H. **(**2004**).** Yeast shuttling SR proteins Npl3p, Gbp2p, and Hrb1p are part of the translating mRNPs, and Npl3p can function as a translational repressor. Mol Cell Biol 24, 10479–10491. 10.1128/MCB.24.23.10479-10491.2004 15542855 PMC529038

[r46] Wu H. I. Brown J. A. Dorie M. J. Lazzeroni L. Brown J. M. **(**2004**).** Genome-wide identification of genes conferring resistance to the anticancer agents cisplatin, oxaliplatin, and mitomycin C. Cancer Res 64, 3940–3948. 10.1158/0008-5472.CAN-03-3113 15173006

[r47] Zimmermann T. Zeizinger M. Burda J. V. **(**2005**).** Cisplatin interaction with cysteine and methionine, a theoretical DFT study. J Inorg Biochem 99, 2184–2196. 10.1016/j.jinorgbio.2005.07.021 16183131

[r48] Zitomer R. S. Hall B. D. **(**1976**).** Yeast cytochrome *c* messenger RNA. *In vitro* translation and specific immunoprecipitation of the *CYC1* gene product. J Biol Chem 251, 6320–6326. 185210

